# Sub-Typing of Extended-Spectrum-β-Lactamase-Producing Isolates from a Nosocomial Outbreak: Application of a 10-Loci Generic *Escherichia coli* Multi-Locus Variable Number Tandem Repeat Analysis

**DOI:** 10.1371/journal.pone.0083030

**Published:** 2013-12-31

**Authors:** Nahid Karami, Lisa Helldal, Christina Welinder-Olsson, Christina Åhrén, Edward R. B. Moore

**Affiliations:** 1 Department of Clinical Microbiology, Sahlgrenska University Hospital and Department of Infectious Diseases, Institute of Biomedicine, Sahlgrenska Academy of the University of Gothenburg, Göteborg, Sweden; 2 Infection Control, Sahlgrenska University Hospital and The Swedish Strategic Programme against Antibiotic Resistance (STRAMA), Västra Götaland Region, Göteborg, Sweden; Queens University Belfast, Ireland

## Abstract

Extended-spectrum β-lactamase producing *Escherichia coli* (ESBL-*E. coli*) were isolated from infants hospitalized in a neonatal, post-surgery ward during a four-month-long nosocomial outbreak and six-month follow-up period. A multi-locus variable number tandem repeat analysis (MLVA), using 10 loci (GECM-10), for ‘generic’ (*i.e.*, non-STEC) *E. coli* was applied for sub-species-level (*i.e.*, sub-typing) delineation and characterization of the bacterial isolates. Ten distinct GECM-10 types were detected among 50 isolates, correlating with the types defined by pulsed-field gel electrophoresis (PFGE), which is recognized to be the ‘gold-standard’ method for clinical epidemiological analyses. Multi-locus sequence typing (MLST), multiplex PCR genotyping of *bla*_CTX-M_, *bla*_TEM_, *bla*_OXA_ and *bla*_SHV_ genes and antibiotic resistance profiling, as well as a PCR assay specific for detecting isolates of the pandemic O25b-ST131 strain, further characterized the outbreak isolates. Two clusters of isolates with distinct GECM-10 types (G06-04 and G07-02), corresponding to two major PFGE types and the MLST-based sequence types (STs) 131 and 1444, respectively, were confirmed to be responsible for the outbreak. The application of GECM-10 sub-typing provided reliable, rapid and cost-effective epidemiological characterizations of the ESBL-producing isolates from a nosocomial outbreak that correlated with and may be used to replace the laborious PFGE protocol for analyzing generic *E. coli*.

## Introduction

Antibiotic multi-resistant *Enterobacteriaceae* with plasmid-mediated resistance mechanisms, such as extended-spectrum β-lactamases (ESBLs) and carbapenemases, are increasingly important causes of community-acquired and nosocomial infections, responsible for expanding patient morbidity and mortality [1]. Of particular concern are the hyper-endemic multi-resistant strains of *Enterobacteriaceae*, particularly *Escherichia coli* and *Klebsiella pneumoniae*, detected world-wide. *E. coli* serotype O25b and sequence type (ST) 131, determined by multi-locus sequence typing (MLST), *i.e.*, strain type O25b-ST131 with well-known virulence properties, is reaching epidemic proportions in many regions world-wide [2], [3]. This strain has been associated predominantly with the ESBL type CTX-M-15, which is the most prevalent ESBL world-wide [4], although additional β-lactamase types increasingly are being reported, including the NDM-1 gene conferring carbapenem resistance [5]. With the rise in antibiotic resistance, the World Health Organization (WHO) has forewarned of the advent of infectious diseases for which no antibiotic treatment will be available (www.who.int/world-health-day/2011). The increasing dearth of new anti-microbial agents in development is leaving few options available for effective treatment of infectious disease. Rapid and reliable diagnoses of infectious bacterial agents, including strain and resistance types, are increasingly important for recognizing multi-resistance and assessing the risk of potential outbreaks.

Molecular subspecies-level typing (*i.e.*, sub-typing) of bacterial strains is imperative for effective epidemiological surveillance of bacteria exhibiting virulence and antibiotic multi-resistance [6]. The ability to differentiate isolates through high-resolution methods is essential for defining the strains that are spreading through epidemic outbreaks and those occurring as sporadic infections or through asymptomatic carriage in hospitals and community settings. This is particularly important for the hyper-endemic strains, such as O25b-ST131. Pulsedfield gel electrophoresis (PFGE) is acknowledged to be the ‘gold standard’ for the typing of strains of a number of bacterial species, including *E. coli*, and is used widely in clinical settings [6]. Unfortunately, the PFGE protocol is time-consuming and labour intensive, requiring rigorous standardization and a high degree of subjective interpretation for direct comparisons over time and between different laboratories. Thus, there is a demand for alternative, more portable sub-typing methods, but with the same level of resolution as PFGE, for differentiating infectious isolates. The multi-locus variable number tandem repeat analysis (MLVA) is a high-resolution method, based upon targeted, PCR-amplified, bar-coded ‘fingerprints’ of tandem repeat sequences distributed throughout the bacterial genome. A particular MLVA protocol has been used widely for strain-typing shiga-toxin-producing *E. coli* (STEC) isolates [7], [8], but was not applicable for differentiating other *E. coli* types, *i.e.*, non-STEC *E. coli* isolates and strains. A new ‘generic’ *E. coli* MLVA, using 10-loci (GECM-10) has been developed recently for differentiating all *E. coli* types [9], [10]. Currently, only two studies have applied GECM-10 for *E. coli* strain typing; one study investigated globally disseminated ESBL-*E. coli*, compared with MLST profiling [11] and the other analyzed STEC isolates, compared with PFGE profiling [12]. The ‘generic’ *E. coli* MLVA assay has never been applied previously for the analysis of an outbreak of pathogenic strains of *E. coli*, such as strain type O25b-ST131. The study reported here is the first that evaluates the GECM-10 profiling protocol for typing ESBL-*E. coli* strains, in comparison with PFGE and MLST, for investigating an epidemiologically well-defined polyclonal ESBL-*E. coli* nosocomial outbreak.

## Materials and Methods

### Bacterial strains and patients

ESBL-*E. coli* were isolated from 28 infants, ages 1–72 months (median age 4 months), hospitalized in a neonatal post-surgery ward at Sahlgrenska University Hospital, Gothenburg, Sweden, between September 2008 to June 2009. This period included an undetected outbreak period from September–December, according to subsequent epidemiological investigation. All isolates with ESBL associated with clinical infections during this period were saved, as well as those obtained during the follow-up period. When the outbreak was detected in December, all hospitalized infant patients were screened repeatedly (twice weekly), until the outbreak was controlled. During January–June all newly admitted infants were screened at admission and twice weekly during hospitalization. The only new cases were those detected at admission. Additionally, on request, more than 100 infant patients hospitalized September–December 2008 submitted samples in early January 2009.

**Figure 1:**
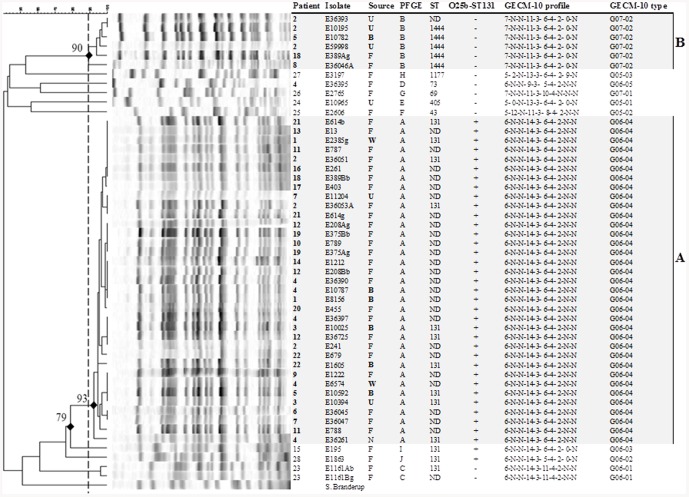
Dendrogram of PFGE profiles correlated with GECM-10 profiles of ESBL-*E. coli* isolates. The relationships between ESBL-*E. coli*, isolated from infant patients during a hospital outbreak and subsequent follow-up period, with the determined PFGE clusters and corresponding GECM-10 types and O25b-ST131 strain detection. Infant patients recognized to be part of the outbreak are indicated in bold type. The PFGE clusters A and B include isolates responsible for the outbreak and are indicated with light grey shading. The sources of isolates are: U = urine; B = blood; F = faeces; W = wound; and N = nasopharynx. The bold type indicates infectious isolates from clinical samples; the remaining isolates are from screening samples. Isolates from each PFGE group were selected for MLST analysis.

Isolates (n = 50) were obtained from urine (n = 6), blood (n = 6), wound (n = 2), nasopharynx (n = 1) and stool (n = 35) samples; 1–6 isolates/child were included in the study of which 13 isolates were derived from clinical samples (Figure 1).

### Identification of isolates and antimicrobial susceptibility testing

Clinical isolates were selected and tested according to routine practice. Samples obtained through patient screening were plated on selective media for Gram-negative bacteria, in the presence of cefuroxime. All cephalosporin-resistant isolates with differing morphologies in a sample were tested further. The isolates were identified as *E. coli*, using conventional biochemical tests. Susceptibilities to tobramycin, thrimethoprim-sulphamethoxazol and/or thrimethoprim, ciprofloxacin, meropenem, and to cephalosporins, were determined, according to the Swedish Reference Group for Antibiotics (SRGA), using the disc diffusion method at the time (http://www.nordicast.org). Cephalosporin-resistant isolates were screened for the ESBL phenotype, using the double-disc diffusion test, with cefotaxime, ceftazidime and cefepime and clavulanic acid as the inhibitory substances [13].

### Genotypic detection of *bla*_CTX-M_, *bla*_TEM_, *bla*_OXA_, *bla*_SHV_

All *E. coli* isolates characterized phenotypically as ESBL-positive were analysed further, using a multiplex PCR assay for detecting CTX-M-, TEM-, OXA- and SHV- β-lactamase genes and for CTX-M phylo-groups, using a real-time TaqMan multiplex PCR assay [14], [15]. Strains positive for ESBLs were included as controls in all PCR assays. CTX-M-, TEM- and OXA- β-lactamase genes were amplified by PCR, using primers and protocols published previously [16], [17]. Individual PCR-amplifications and sequencing of all genes were performed, as described previously [18]. The raw trace files were edited and contig sequences were generated, using the BioNumerics version 6.6 software (Applied Maths NV, Sint-Martens-Latem, Belgium).

### Pulsed-field gel electrophoresis (PFGE)

Isolates were sub-typed by PFGE profiling, following extraction of genomic DNA and digestion with *Xba*I restriction enzyme [19] and as described previously [20]. *Xba*I-digested DNA from *Salmonella* serotype Braenderup H9812 was included as a normalization standard on every gel. DNA band patterns were analyzed, using the BioNumerics software version 6.6 with the Dice coefficient for calculating pair-wise similarities and the UPGMA algorithm for constructing dendrograms of estimated relatedness. Position tolerance and optimization were both set at 1.0%. Strains were designated to be indistinguishable if their electrophoresis profile similarities were 100% and considered to be closely related if their similarities were ≥90% [6].

### Multi-locus sequence typing (MLST)

MLST was performed, according to the method of the *E. coli* MLST database website [21]. Seven house-keeping genes: *adk*, *fumC*, *gyrB*, *icd*, *mdh*, *purA*, and r*ecA* were targeted for PCR-amplification and sequencing. The sequences were submitted to the MLST database for *E. coli* and the respective sequence types were determined (http://mlst.ucc.ie/mlst/dbs/Ecoli).

### Identification of O25b-ST131 strain by PCR

Detection of the O25b-ST131 strain-type was performed, using an O25b-ST131 allele-specific *pabB* PCR [22]. The *adk*-gene was used as the positive PCR control for assessing DNA-quality (http://mlst.ucc.ie/mlst/dbs/Ecoli).

### Generic *E. coli* MLVA using 10 loci (GECM-10)

GECM-10, targeting nine tandem sequence repeats (CVN001, CVN002, CVN003, CVN004, CVN007, CVN014, CVN015, CVN016 and CVN017) and one regularly interspersed short palindromic repeat (CCR001) was performed, using four PCR assays, with HotStarTaq Master Mix (Qiagen, Hilden, Germany) and multiple dye-labelled amplification primers [9], [10].

The first PCR contained primers for the CVN003 and CVN014 loci, the second PCR for CVN001, CVN004, CVN007 and CVN015, the third PCR for CCR001, CVN016 and CVN017 and the fourth PCR was a single PCR for the CVN002 loci. The final concentrations of the PCR primers were slightly modified from those previously described [9], [10]. The concentrations of primers for the CVN0001, CVN002, CVN007, CVN014, CVN 016 and CVN 017 loci were 0.2 µM per reaction and 0.4 µM, 0.8 µM, 1.2 µM and 0.12 µM, respectively, for the CCR001, CVN003, CVN004 and CVN15 loci. After PCR-amplifications, the PCR-products were pooled; 2.5 µl of multiplex PCR no. 1, 2 µl of PCR product no. 4 and 30 µl dH_2_O. To 1.0 µl of this pooled solution, 0.5 µl of the Geneflo-625 TAMRA (CHIMERx) internal size standard was added with 12 µl formamide and electrophoresed, using an ABI-310 Genetic Analyzer (Applied Biosystems) as published [9]. The products of PCR no. 2 (1.0 µl) and PCR no. 3 (1.0 µl) were added to 50 µl dH_2_O, in separate solutions, and electrophoresed, as described above. The solution with PCR no. 4 was mixed with Genescan-Liz600 (Applied-Biosystems, foster city, CA, USA) instead of Geneflo-625 TAMRA. For all loci, each peak was identified, according to dye-label and size. Based on the PCR-product size, each allele number was calculated (Table 1), as described previously [10].

**Table 1:** Identifications of the 10 detected ESBL-*E. coli* GECM-10 types, designated by the allele numbers of each GECM profile and corresponding PFGE types.

GECM-10 type	GECM-10 profile	PFGE type
G05-01	5 - 0 - N -13 - 3 - 6 - 4 - 2 - 0 - N	E
G05-02	5 -12 - N -11 - 3 - 8 - 4 - 2 - N - N	F
G05-03	5 - 2 - N -13 - 3 - 6 - 4 - 2 - 9 - N	H
G06-01	6 - N - N -14 - 3 -11 - 4 - 2 - N - N	C
G06-02	6 - N - N -14 - 3 - 5 - 4 - 2 - 0 - N	J
G06-03	6 - N - N -14 - 3 - 6 - 4 - 2 - 0 - N	I
G06-04	6 - N - N -14 - 3 - 6 - 4 - 2 - N - N	A
G06-05	6 - N - N - 9 - 3 - 5 - 4 - 2 - N - N	D
G07-01	7 - N - N -11 - 3 -10 - 4 - N - N - N	G
G07-02	7 - N - N -11 - 3 - 6 - 4 - 2 - 0 - N	B

GECM-10 type numbering: ‘G’ and the allele number for the first locus, followed by the designated number of the allelic profile in the GECM-10 database at Sahlgrenska University Hospital. The loci in the profiles are presented in the following order: CVN001; CVN002; CVN003; CVN004; CVN007; CVN014; CVN015; CCR001; CVN016; and CVN017 [10]. Absent loci, *i.e.*, loci for which a PCR-amplification product was not generated, were designated by ‘N’ (i.e., ‘no product’). Positive PCR-amplification products that contained no repeats were designated by ‘0’ (*i.e.*, ‘null’).

## Results and Discussion

### Identification of ESBL-*E. coli*

The 50 ESBL-*E. coli* isolates were obtained from 28 infants, of which 41 isolates (from 21 children) were concluded, from the epidemiological investigation, to constitute the outbreak of two distinct *E. coli* strains of PFGE type A/GECM-10 type G06-04 and PFGE type B/GECM-10 type G07-02 (Table 2, Figure 1). The infant patients carried either or both of these two strains, four infants (no. 2, 4, 5 and 18) carried multiple strains isolated at the same or at subsequent sampling occasions. The outbreak went undetected for several months, partly due to its polyclonal nature. The long, unrevealed outbreak period may have contributed to the number of strain variants detected. Additionally, each of three infants (no. 4, 23 and 24) hospitalized for a short time during the outbreak period carried ESBL-*E. coli* strains of unique PFGE types (C, D and E), either alone or in combination with the outbreak strains. Five infants (no. 15, 25, 26, 27 and 28) were positive for ESBL-*E. coli* in stool samples at admission during the follow-up period and, thus, were not considered to be part of the outbreak. All of these infants carried isolates of unique PFGE-types (F-J). All 50 *E. coli* isolates were divided into 16 groups, according to their PFGE and GECM-10 types, β-lactamase gene carriage and antibiotic susceptibility patterns, as shown in Table 2.

**Table 2:** GECM-10-types and the respective PFGE-types, β-lactamase genotypes and antibiotic resistance profiles for the ESBL-*E.coli* isolates analysed.

GECM-10 type	PFGE type	No. isolates (patients)	β -lactamase genes^a^			Antibiotic resistance^b^
G06-04	A	25 (15)	CTX-M-15	OXA-1	TEM-1b	TOB - TMP
G06-04	A	8 (5)	CTX-M-15	-	TEM-1b	TMP
G06-04	A	2 (2)	CTX-M-15	-	TEM-1b	TOB - TMP
G06-03	I	1	CTX-M-15	OXA-1	-	TOB - CIP
G06-02	J	1	CTX-M-15	OXA-1	-	TOB - TMP - CIP
G06-01	C	2 (1)	CTX-M-14	-	TEM-1b	TMP
G06-05	D	1	CTX-M-15	OXA-1	-	TOB
G07-02	B	1	CTX-M-15	OXA-1	-	TOB
G07-02	B	1	CTX-M-15	OXA-1	TEM-1b	TOB - TMP
G07-02	B	1	CTX-M-15	-	TEM-1b	TOB - TMP
G07-02	B	2 (1)	CTX-M-15	-	TEM-1b	TMP
G07-02	B	1	CTX-M-15	-	-	-
G07-01	G	1	CTX-M-15	-	-	TMP - CIP
G05-01	E	1	CTX-M-15	OXA-1	-	TOB - TMP - CIP
G05-03	H	1	CTX-M-15	-	TEM-1b	CIP
G05-02	F	1	CTX-M-15	OXA-1	TEM-1b	TOB - TMP - CIP

^a^ One isolate from each group was selected for ß-lactamase gene sequencing. If a particular group included isolates from multiple sources, the ß-lactamase genes of one isolate from each source was included; in total, the ß-lactamase genes of 23 isolates were sequenced.

^b^ Phenotypic resistance to tobramycin (TOB), trimethoprim-sulphamethoxazol and/or thrimethoprim (TMP) and ciprofloxacin (CIP). All isolates were resistant to third generation cefalosporins; all isolates were susceptible to meropenem.

### β-lactamase genotyping and antibiotic resistance profiling

The profiles for β-lactamases, including the OXA, CTX-M, TEM and SHV genes, and the antibiotic resistance patterns of the isolates were determined (Table 2). Importantly, the strain type could not be correlated or predicted by the presence of particular individual β-lactamase genes or combination of genes. The most predominant β-lactamase combinations, *i.e.*, CTX-M-15, TEM-1b and OXA-1, (27 isolates) and CTX-M-15 and TEM-1b (14 isolates), were represented in three different PFGE strain types. Thus, similar β-lactamase gene carriage combinations were observed in isolates of different PFGE-types, and vice versa. The low discriminatory value of typing β-lactamase genes in this outbreak investigation was evident, particularly when a given CTX-M, *i.e.*, CTX-M-15, has become so predominant in a region [23].

The resistance to routinely tested antibiotics demonstrated similar resistance patterns for isolates of different PFGE-types and isolates of the same PFGE-types exhibited different resistance patterns, even for repeat isolates from the same patient. Therefore, relying on antibiotic resistance profiling as an indication of an outbreak, which has been a commonly used surveillance strategy, can be recognized as potentially misleading; such practice delayed the recognition of this outbreak by months. Furthermore, the potential for differentiating isolates by antibiotic resistance profiling can be expected to be further reduced with the increase in multi-resistance in *E. coli* isolates. The combination of β-lactamase genotyping and antibiotic resistance profiling as tools for the recognition and sub-typing of ESBL-*E. coli* isolates was not more reliable than either of the two methods alone (Table 2).

### Strain-typing of ESBL-*E. coli* isolates by PFGE

Ten PFGE types (A to J) were detected among the 50 ESBL-*E. coli* isolates (Figure 1, Table 2). The PFGE type A isolates, comprising 35 isolates from 20 different patients, exhibited ≥93% profile similarity. They were epidemiologically related and were collected only from children hospitalized during the outbreak period, suggesting that the PFGE type A was the likely predominant outbreak strain. Isolates of PFGE type B included six isolates from four children, also hospitalized during the outbreak period.

Isolates of PFGE types C to E were obtained from children hospitalized during the outbreak period, the isolates were detected in stool samples, each from only one child. The strains of type F to J were isolated from children at admission, during the follow-up period. Thus, the isolates C-J were considered to be community-acquired strains carried by these children and not part of the outbreak, demonstrating the importance of epidemiological data being available in the assessment of outbreaks.

### Strain-typing of ESBL-*E. coli* isolates by MLST

Representative isolates (n = 23) from each GECM-10 and PFGE type, as well as selected isolates carrying each of different β-lactamase gene combinations, antibiotic resistance profiles and different sampling sources were analyzed by *E. coli* MLST [21]. MLST detected seven different sequence types (Figure 1) and could not differentiate between PFGE types A, C, I and J, despite the similarity index being as low as 62%. This demonstrates the limited value for MLST as a reliable sub-typing method for *E. coli* outbreak investigations. However, for long-term surveillance, especially for heterogeneous clones, such as those of ST131, the isolate sequence type is important to monitor. The O25b-ST131 strain type was missed when relying only on PFGE typing data, as has also been reported from the United Kingdom [24].

### Detection of O25b-ST131 strains by PCR

All isolates of PFGE types A, I and J belonged to the pandemic strain type O25b-ST131, while none of the other PFGE-types did (Figure 1). As demonstrated by other reports, this strain type is genotypically diverse [25]. The isolates of PFGE type A exhibited profile similarities of 93% or greater, while PFGE types I and J were delineated at <79% similarity to strains of PFGE type A. However, the outbreak demonstrated the propensity of PFGE type A to be transmitted between individuals. The global predominance of O25b-ST131 may limit the value of the O25b-ST131 PCR assay for outbreak investigations, other than as an initial screening method for ESBL-*E. coli* and could not differentiate outbreak isolates from non-outbreak community acquired isolates in this study.

### Strain-typing of ESBL-*E. coli* isolates by GECM-10

Ten distinct GECM-10 strain types that differed by, at least, one allele were identified among the 50 ESBL-*E. coli* isolates (Table 1 and 2, Figure 1). Loci CVN003 and CVN017 were absent (N) in all studied isolates and CVN007 and CVN015 loci shared the same allele type in all of them. The greatest variations in repeats were seen within loci CVN004 and CVN014. Each unique GECM-10 type corresponded to a unique PFGE type and vice versa. All isolates in PFGE cluster A shared the same GECM-10 profile as did all isolates in PFGE cluster B.

All isolates of type ST131 shared the same GECM-10 profile in all but the CVN014 and CVN016 loci. The isolates not belonging to *E. coli* O25b-ST131 differed by a marked number of repeats in locus CNV014, *i.e.*, 11, as compared to five or six repeats observed for isolates of the O25b-ST131 type. Furthermore, the two *E. coli* O25b-ST131 type isolates not belonging to cluster A by PFGE also could be differentiated by GECM-10. CVN016 could be amplified in both isolates, with a PCR-product of 475 bp, although the repeat number was not able to be determined and, thus, was designated as ‘0’ (*i.e.*, ‘null’), whereas no amplifications of the CVN016 locus (N) were observed in isolates of PFGE cluster A (Table 1, Figure 1). The isolate of PFGE type J that was more distantly related to the outbreak isolates of cluster A, according to PFGE profiling, also lacked one repeat in locus CVN014, compared to the type A and I isolates. Both of these isolates were fluoroquinolone resistant, as compared to the type A isolates. Interestingly, this resistance type has been reported previously to exhibit distinct PFGE-profiles within the 131 sequence type [26]. Notably, locus CVN014 is known to be the most variable locus in the GECM-10 assay [27], [28]. CVN016 also has been reported to exhibit variation among ESBL-*E. coli* isolates [10]. Considering the recognized heterogeneity in *E. coli* O25b-ST131 [25], it is not unlikely that the number of tandem repeats may change over time in isolates of this type, particularly in the loci that are known to vary. Variation at a single locus, *e.g.*, through homoplasy, may result in an underestimation of the actual genotypic variation between strains. However, the efficacy of the generic *E. coli* GECM-10 assay is based upon the combinations of highly variable and conserved loci, rather than only single loci. It has the ability to apply the more variable loci, such as CVN014 and CVN016, for high-resolution sub-typing of closely related isolates, such as those in an outbreak situation, as well as more distantly related isolates and those belonging to distinct serotypes, by using less variable loci [28]. This novel application of GECM-10 for analyzing outbreak isolates and comparison with PFGE in this study has demonstrated the discriminatory power of this new MLVA protocol that may be applicable for replacing the PFGE method in the clinical laboratory. There are many studies reported in the literature on using MLVA typing for analyzing EHEC and STEC isolates, rather than ‘generic’ *E. coli*. It is important to recognize that the targeted loci of the STEC-MLVA and *E. coli*-GECM-10 are different. To date, the generic *E. coli* MLVA, using 10-loci, has never before been applied and compared to PFGE (*i.e.*, the ‘gold-standard for clinical sub-typing) for analyzing ESBL-*E. coli* isolates from an outbreak. One study showed a high discriminatory power of GECM, using seven loci, compared to PFGE results, for investigating generic *E. coli* isolates from patients with urinary tract infections [29]. In short-term investigations, like the outbreak of this study, it is important to increase the resolution among isolates, favoring the use of GECM-10 rather than GECM with only seven loci.

## Conclusion

The increasing prevalence of ESBL-producing strains of *E. coli*, as well as *E. coli* producing carbapenemases, such as NDM-1 and KPC-2, will likely lead to outbreaks in hospital settings and generate increasing demands for rapid sub-typing methods with high and clinically-relevant discriminatory power. The world-wide proliferation of particular strain types, such as O25b-ST131, adds additional relevance for considering the methods to be used. The epidemiologically well-characterized polyclonal outbreak of this study, including a subsequent follow-up period of monitoring patients to confirm that the outbreak had ceased, has provided the opportunity to evaluate alternative methods for sub-typing ESBL-*E. coli* in comparison with PFGE. The results of this study demonstrated the limited values of ESBL-gene typing and antibiotic resistance profiling alone, as well as in combination. With the increasing prevalence of O25b-ST131, PCR assays for this strain type most likely will be of decreasing epidemiological value, except as a possible initial screening method, depending on the local community prevalence of this strain.

GECM-10 has been demonstrated to provide excellent discrimination of isolates, comparable to that of PFGE. The discriminatory power of GECM-10 should be further analyzed among the closely related isolates of pandemic strains, such as type O25b-ST131, and unrelated isolates occurring sporadically, and compared to PFGE typing, which is recognized as the so-called, ‘gold standard’ method, before application of the GECM-10 assay in the routine strain-typing of clinical isolates. With respect to data portability, the use of fragment profiling by GECM-10 to generate a ‘bar-code’ is markedly advantageous. Furthermore, this outbreak investigation demonstrated the importance of the relevant epidemiological data in evaluating strain-typing methods to be used for outbreak detection.
